# Reduced P300 amplitude during retrieval on a spatial working memory task in a community sample of adolescents who report psychotic symptoms

**DOI:** 10.1186/1471-244X-13-125

**Published:** 2013-05-01

**Authors:** Caroline Rawdon, Jennifer Murphy, Mathieu M Blanchard, Ian Kelleher, Mary C Clarke, Fergal Kavanagh, Mary Cannon, Richard AP Roche

**Affiliations:** 1 Department of Psychology, National University of Ireland, Maynooth, Co. Kildare, Ireland; 2 Department of Psychiatry, Royal College of Surgeons in Ireland, Dublin, Ireland; 3 Department of Psychiatry, Beaumont Hospital, Dublin 9, Ireland

**Keywords:** Spatial working memory, Retrieval, Adolescence, Psychotic symptoms, Event-related potentials, P300, Sternberg paradigm

## Abstract

**Background:**

Deficits in working memory are widely reported in schizophrenia and are considered a trait marker for the disorder. Event-related potentials (ERPs) and imaging data suggest that these differences in working memory performance may be due to aberrant functioning in the prefrontal and parietal cortices. Research suggests that many of the same risk factors for schizophrenia are shared with individuals from the general population who report psychotic symptoms.

**Methods:**

Forty-two participants (age range 11–13 years) were divided into those who reported psychotic symptoms (*N* = 17) and those who reported no psychotic symptoms, i.e. the control group (*N* = 25). Behavioural differences in accuracy and reaction time were explored between the groups as well as electrophysiological correlates of working memory using a Spatial Working Memory Task, which was a variant of the Sternberg paradigm. Specifically, differences in the P300 component were explored across load level (low load and high load), location (positive probe i.e. in the same location as shown in the study stimulus and negative probe i.e. in a different location to the study stimulus) and between groups for the overall P300 timeframe. The effect of load was also explored at early and late timeframes of the P300 component (250-430 ms and 430-750 ms respectively).

**Results:**

No between-group differences in the behavioural data were observed. Reduced amplitude of the P300 component was observed in the psychotic symptoms group relative to the control group at posterior electrode sites. Amplitude of the P300 component was reduced at high load for the late P300 timeframe at electrode sites Pz and POz.

**Conclusions:**

These results identify neural correlates of neurocognitive dysfunction associated with population level psychotic symptoms and provide insights into ERP abnormalities associated with the extended psychosis phenotype.

## Background

In recent years the importance of extending research in the area of psychosis to include those who report psychotic symptoms in the general population has been highlighted as a new approach to identifying individuals at increased symptomatic risk for developing a psychotic disorder [1]–[3]. Self-reports of psychotic symptomatology in adolescence have been found to be significantly associated with the development of a psychotic disorder in adulthood [4], [5], and the clinical psychosis phenotype (schizophrenia and other psychotic disorders) and non-clinical psychosis phenotype (individuals who do not have psychotic disorders but who report experiencing psychotic symptoms) have been shown to share many of the same risk factors [6]. In addition to increased risk for psychotic disorder, recent research has demonstrated that the majority of individuals in the population who report psychotic symptoms have at least one (non-psychotic) Axis-1 disorder [7], a finding which is in keeping with research in clinic-presenting young people with at risk mental states [8]–[11], who are considered to be at ‘ultra high risk’ for psychosis [12]. In fact, community-based research on young people who report psychotic symptoms has shown that this group is at high risk of multimorbidity [7] (that is, the presence of multiple co-occurring psychiatric disorders) and poorer illness course than individuals with psychopathology who do not report psychotic symptoms [13]. Individuals with psychopathology who report psychotic symptoms have also been demonstrated to be at high risk for severe suicidal behaviour [14], [15], making this group important both in terms of psychosis risk as well as in terms of risk for severe non-psychotic psychopathology.

Working memory deficits have been hypothesised as a core deficit in schizophrenia [16]. Wood et al. [17] state that working memory may represent a neurocognitive trait marker for schizophrenia, as it is considerably impaired throughout the illness and involves neural circuits deemed dysfunctional in the disorder. Aleman et al. [18] report that memory impairments do not seem to be modality specific and that clinical variables such as medication, duration of illness, patient status, severity of psychopathology and positive symptoms do not appear to influence the magnitude of memory impairment. In addition, no evidence of progressive decline in memory with duration of illness was observed in their meta-analysis, suggesting that memory impairment may be a trait rather than a state characteristic.

Both verbal and visuospatial working memory impairments have been observed in schizophrenia and first episode psychosis (FEP) patients [19]–[21] and groups defined as genetically at-risk of developing schizophrenia [22]–[25]. Similar impairments have been observed in groups considered clinically at-risk for psychosis, such as prodromal/Ultra High-Risk (UHR)/At-Risk Mental State (ARMS) groups [26]–[28], as well as groups who present putative antecedents of schizophrenia including psychotic-like experiences [29]. Visuospatial working memory deficits have also been reported in adolescent-onset schizophrenia [30], [31]. Most recently, deficits in spatial working memory have been reported in a community-based sample of adolescents reporting psychotic symptoms [32] and who met formal criteria for prodromal risk syndromes [33].

In a meta-analysis of the literature, Bramon et al. [34] state that the P300 waveform has been conceptualised as the physiological correlate of a working memory update of changes in the environment [35], or as an index of allocation of attentional resources [36]. According to Marchand et al. [37], many ERP studies of working memory have employed a modified version of the Sternberg task in which participants are presented with a set of digits/letters to memorise, followed by a “probe” digit. The participant must indicate if the probe was part of the original memory set (positive probe) or not (negative probe). Behaviourally, studies have shown that accuracy on working memory tasks decreases and reaction time increases as the memory load (number of items to be remembered) increases [38].

In a summary of previous research, Marchand et al. [37] state that the most consistent finding in the literature is the elicitation of a sustained parietal positivity (i.e. P300) to the probe (irrespective of whether the probe type is positive or negative) with an increase in latency as memory set size increases. Bledowski et al. [39] propose that the P3b component can be separated into two subcomponents; they propose that the early P3b subcomponent is related to stimulus evaluation processes, whereas the later P3b subcomponent reflects memory search processes in the ventrolateral prefrontal cortex, which access a posterior parietal storage buffer. Polich [40] proposes that the P3b originates from temporal-parietal activity associated with attention and appears to be related to subsequent memory processes.

Imaging studies of working memory have largely focused on the functioning of the prefrontal and the parietal cortices [PFC and PC; [41]–[43]. Studies have suggested a role for both the dorsolateral and ventrolateral prefrontal cortex (dlPFC and vlPFC) in visuospatial working memory [43], [44]. Working memory deficits in schizophrenia patients and those genetically at-risk have been linked to abnormal brain activity in the PFC [23]. Studies have reported both hypoactivation [45], [46] and hyperactivation [47]–[50] of areas of the PFC in patients with schizophrenia. Keshavan et al. [51] reported decreased activity in the dlPFC and inferior PC in offspring of individuals with schizophrenia, while Broome et al. [52] reported decreased activity in the medial PFC and right precuneus in a group of ARMS participants during spatial working memory tasks.

A reduction of the P300 component in schizophrenia patients is one of the most consistent findings in the literature [34]. Galletly et al. [53] used a Two-In-A-Row (TIAR) auditory oddball task which they argue necessitates the repeated updating of target identity, thus requiring stimuli to be maintained in working memory. They reported reduced P300 amplitude in schizophrenia participants relative to controls for both non-target and target tones in their task. A reduction of the amplitude of the P3b component during encoding and retrieval on a visual working memory task has been reported in patients with early-onset (adolescent) schizophrenia [53]. Haenschel et al. [54] propose that the early posterior P3b peak observed in their study may reflect stimulus evaluation while the later P3b peak may reflect consolidation during encoding and template matching during retrieval [39], [53], [55].

To date, no electroencephalography (EEG) studies of spatial working memory have been carried out with adolescents who report psychotic symptoms. The present paper investigates the electrophysiological correlates of spatial working memory in a group of adolescents who reported psychotic symptoms compared to a matched group of controls. This was achieved using a Spatial Working Memory Task developed for use with an adolescent population based on the classic Sternberg working memory paradigm [38].

We hypothesised that there would be differences in ERP amplitude between those participants who reported psychotic symptoms and the control group for the P300 component in line with amplitude reductions reported in schizophrenia patients and adolescent-onset schizophrenia [53], [54]. While increases in latency and sustained amplitude of the P300 component were predicted for all participants as memory load increased, it was hypothesised that P300 amplitudes would be reduced in the psychotic symptoms group compared to the control group.

## Methods

### Participants

Participants were recruited from the two most senior classes in primary schools in north Co. Dublin and counties Kildare and Meath. Details of the recruitment procedure have been previously reported [56]. Forty-two participants (21 male; age range 11–13 years; mean age = 12.14 years) completed the Spatial Working Memory Task after completing a clinical interview using the Kiddie- Schedule for Affective Disorders and Schizophrenia for School Aged Children (6–18 Years) – Present and Lifetime Version [K-SADS-PL; [57]. Seventeen participants reported psychotic symptoms (7 male; age range 11–13 years; mean age = 12 years; 1 left handed). Twenty-five participants reported no psychotic symptoms (14 male; age range 11–13 years; mean age = 12.24 years; 0 left handed). Participants gave written informed assent, and parental consent was obtained before participation in the study. All participants had normal or corrected-to-normal vision and no previous neurological disorders or brain injuries or family history of psychotic illness. This research was approved by the Ethics Committees of Beaumont Hospital and the National University of Ireland, Maynooth.

### Assessment of psychotic symptoms and general functioning

The psychosis section from the K-SADS-PL [57] was used to probe psychotic symptoms such as hallucinations and delusions. The most commonly reported psychotic symptoms in the K-SADS interview were auditory hallucinations, which were often accompanied by some form of delusional beliefs. Auditory hallucinations that were deemed as clinically significant included voices commenting on behaviour, a voice giving commands, voices conversing, whispering voices and voices at varying volumes where the words cannot clearly be distinguished by the individual. Clinically significant delusions included bizarre ideas of being watched (e.g., by police using hidden security cameras in the home), unfounded ideas that others are saying negative things about the individual (which are distinguished from normal teenage self-consciousness), and a belief that ghosts are communicating directly with the individual. Extensive notes were recorded during the clinical assessment and symptoms were rated as psychotic based on a subsequent consensus meeting between three mental health professionals, blind to all information other than the psychosis section of the K-SADS-PL. Following the clinical interview a Children’s Global Assessment Scale score [C-GAS; [58] was assigned to assess each participants’ current level of functioning. The Wide Range Achievement Test–4 [WRAT-4; [59] was administered as a measure of scholastic ability.

### Spatial working memory task

A Spatial Working Memory Task developed for use with an adolescent population based on the classic Sternberg working memory paradigm [28]. The task was written and presented in E-Prime E-Studio®. Participants were presented with a study array showing a number of target stimuli in fixed placeholders. The task consisted of 128 trials in total. Each block contained 32 test trials in which participants made a button press response, via a mouse with their index or middle finger, to indicate whether a stimulus was in a correct (positive probe) or incorrect (negative probe) location, respectively.

**Figure 1:**
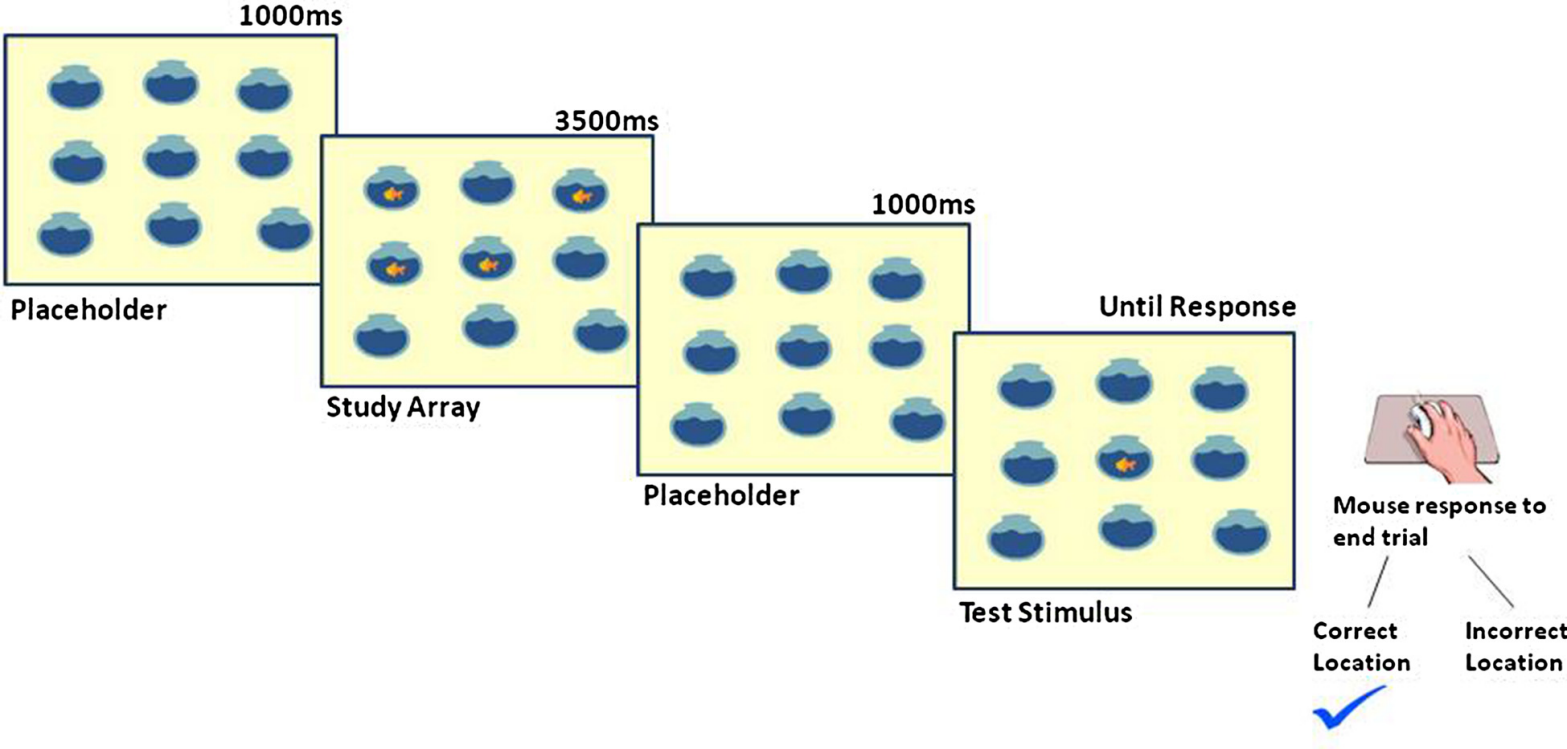
Example from block 1 (low load) of the working memory task displaying a fish bowl placeholder array, followed by the study array, followed by another placeholder array and a test stimulus in a correct location.

Stimuli were either fish in fishbowls or birds in bird cages generated in Microsoft Word using ClipArt (see Figure 1 for an example of a trial). For each block, participants were instructed to remember which placeholders (i.e. fishbowls or bird cages) contained target stimuli (i.e. fish or birds). Subsequently a target stimulus would appear in one of the placeholders and the participants would have to remember whether they had seen a stimulus in that placeholder.

Specifically, participants were initially presented with the blank placeholder array for 1000 ms (which displayed the empty placeholders for that block). Participants were then presented with a study array which displayed a number of target stimuli in a subset of the placeholders. This study array was presented for 3500 ms. The presentation of the study array was then followed by another presentation of the blank placeholder array for a further 1000 ms. Finally, participants were presented with the test stimulus (one target stimulus presented in the placeholder array), which required the participant to make a button press response with their right hand using a mouse which terminated each trial to indicate whether the test stimulus was in a correct (left button) or incorrect (right button) placeholder. For the first half of each block participants viewed one study array, following which 16 probe stimuli using the fish/fishbowl stimuli were presented as test stimuli. The second half of each block contained one study array using the bird/birdcage stimuli, followed by a further 16 probe stimuli.

The task included 4 blocks (2 × low memory load and 2 × high memory load). Blocks 1 and 2 were classed as low memory load, while Blocks 3 and 4 were classed as high memory load. In Block 1, 4 out of 9 placeholders in the study array contained a target stimulus (as in Figure 1). In Block 2, 5 out of 12 placeholders in the study array contained a target stimulus. In Block 3, 6 out of 15 placeholders in the study array contained a target stimulus, while in Block 4, 7 out of 18 placeholders in the study array contained a target stimulus.

**Figure 2:**
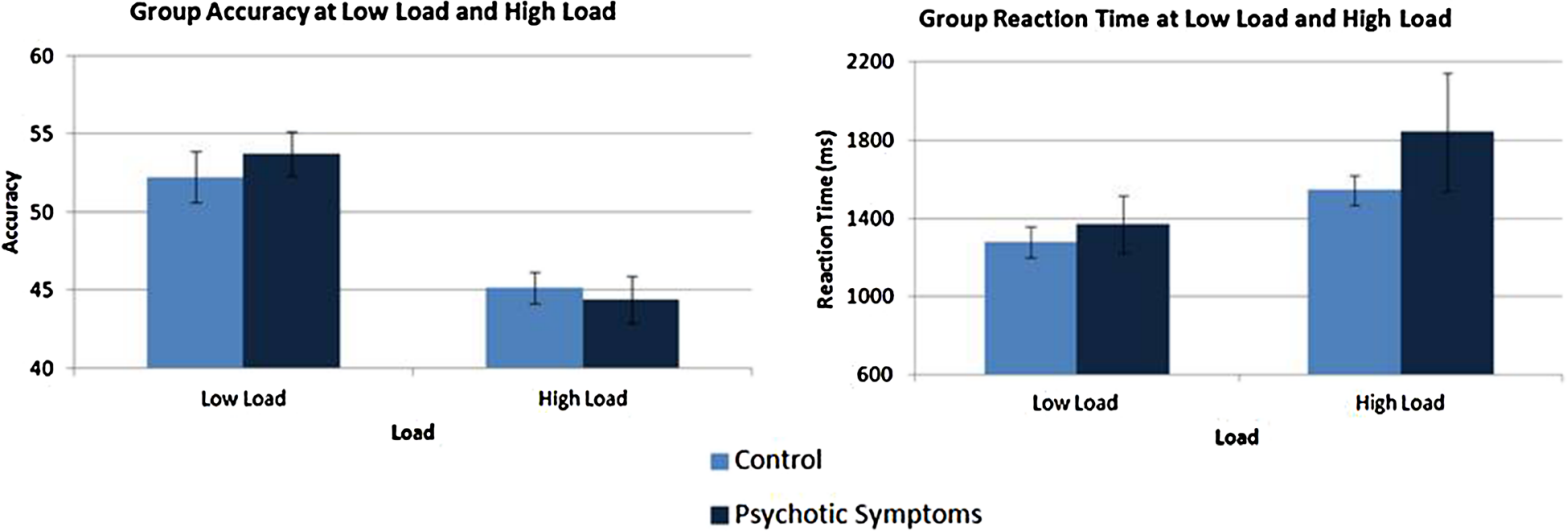
**Top - ERP waveforms showing group differences in mean amplitude for correct responses at Pz and POz.** Bottom - ERP waveforms and corresponding scalp topographic maps (control group) showing group differences in mean amplitude for correct responses at Low Load and High Load at POz.

### Procedure

E-Prime© logged response times for each participant and sent Transistor-Transistor Logic (TTL) triggers to the EEG acquisition PC to allow stimulus presentations (stimulus type) and responses to be logged in real time on the EEG recording. Response times were measured as the time between presentation of the test stimulus and the response, and were recorded for all trials. Response latencies and accuracy were calculated automatically by E-Prime© and average response times were collated in E-Prime© for each block.

EEG data were recorded in microvolts (μV) from 62 scalp sites positioned according to the extended international 10–20 system of electrode placement. The nasion was used as a reference. All impedances were below 10 kΩ. Vertical eye movements (VEOG) were recorded from electrodes located above and below the left eye and horizontal eye movements (HEOG) were recorded from the electrodes positioned at the outer canthus of each eye. Blinks were averaged off-line and a blink reduction algorithm was applied to the data. This algorithm involved automatic artifact correction [60], [61]. Stimulus-locked average ERPs were obtained by averaging the EEG using the test stimulus presentation as the trigger. Scalp sites chosen for further analysis were based on visual inspection of the scalp topographies for the task. ERP waveforms were maximal at midline recording sites for the task, therefore sites Pz and POz were chosen for further analysis of the P300 ERP component. ERP component time windows were chosen based on previous literature and visual inspection of grand averaged waveforms. Stimulus-locked epochs were defined as -100 ms pre-stimulus presentation until 1000 ms after stimulus presentation. Data were averaged for each participant and grand averages for each block were compiled. Only epochs in which participants made correct responses were included for group comparisons of ERP mean amplitude and latency analysis.

### Statistical analyses

Statistical Analyses were carried out using SPSS Statistics version 20 for Windows. Demographic variables were compared using independent-samples t-tests and chi-square analyses. Separate 2×2 repeated measures ANOVA were used to examine differences in mean accuracy scores and mean reaction times (defined in milliseconds - ms) between high and low loads for the overall group. Independent-samples t-tests were used to test between-group differences in mean accuracy and mean reaction times. A 2×4 repeated measures ANOVA was used to compare the number of accepted trials for each group across each block of the task and no between-groups effect was observed [*F*(1, 38) = 0.2, *p* = 0.65]. Separate 2×2×2 mixed factorial ANOVAs were utilised to examine mean amplitude and mean latency differences respectively, with Load Level (low, high) and Location (positive probe, negative probe) as within-subjects variables, and Group (psychotic symptoms group, control) as the between-subjects variable to test for differences at each of the electrode sites chosen for further analysis (Fz, Pz, POz). Independent-samples t-tests were then used to further examine observed between-groups differences. Separate 2×2×2 mixed factorial ANOVAs were used to further examine mean amplitude difference at Pz and POz with Load Level (low, high) and Time (250 ms to 430 ms, 430 ms to 750 ms) as within subjects variables and Group (psychotic symptoms group, control) as the between-subjects variable. Latency was defined as the most positive data point within the specified component timeframe (Early P300 – 250-430 ms) and a 2×2 mixed factorial ANOVA was used to examine the effect of Load (low, high) across Groups (psychotic symptoms group, control). Independent-samples t-tests were then used to further examine observed between-group differences. For each ANOVA, an alpha value of 0.05 was used for main and interaction effects. Greenhouse-Geisser correction was employed where the assumption of sphericity was violated.

## Results

### Demographic comparisons

Groups were compared on age, gender, overall current C-GAS scores from the K-SADS-PL interview schedule [56] and scores on the WRAT-4 [59]. No between-group differences in mean age [*t*(40) = 0.97, *p* = 0.34], gender [*χ*^*2*^(1) = 0.14, *p* = 0.71], handedness (*χ*^*2*^(1) = 0.04, *p* = 0.844) or WRAT-4 scores [*t*(34) = −0.35, *p* = 0.73] – see Table 1.

**Table 1:** Demographic and general functioning details for the control and psychotic symptoms groups

**Variable**	**Overall (*N*** **= 42)**	**Control (*N*** **= 25)**	**Psychotic Symptoms (*N*** **= 17)**	**Result**
**Age**	12.14(0.12)	12.24(0.17)	12(0.17)	*t*(40) = 0.97, *p* = 0.34
**Gender**	21 males	14 males	7 males	*χ*^*2*^(1) = 0.14, *p* = 0.71
**Handedness**	1 left	0 left	1 left	*χ*^*2*^(1) = 0.04, *p* = 0.844
**C-GAS scores**	79.45(2.67)	84.88(2.33)	71.31(5.16)	***t*(38) = 2.4, *p*** **= 0.03**
**WRAT scores**	47.28(1.56)	46.8(2.4)	47.88(1.9)	*t*(34) = −0.35,*p* = 0.73

Age, C-GAS scores and WRAT scores expressed as mean (SEM).

### Behavioural data

No significant differences in overall mean accuracy scores were observed between the control and psychotic symptoms groups [*F*(1, 39) = 0.07, *p* = 0.8]. No interaction effect was observed between load and group. A main effect of load was observed [*F*(1, 39) = 36.73, *p* < 0.0005] with accuracy higher for low load than at high load.

No significant differences in overall mean reaction time scores for correct responses were observed between the control and psychotic symptoms groups [*F*(1, 39) = 0.94, *p* = 0.34]. No interaction effect was observed between load and group. The main effect of load reached statistical significance [*F*(1, 39) = 16.17, *p* < 0.0005], with slower responses for high load.

Due to the presence of large standard error (SEM) in the psychotic symptoms group for high load, the coefficient of variation (standard deviation/mean) was calculated for reaction time data for both groups for both low and high load (see Figure 2). When tested with analysis of variance however, no significant difference was observed between the groups [*F*(1, 39) = 0.84, *p* = 0.37].

**Figure 3:**
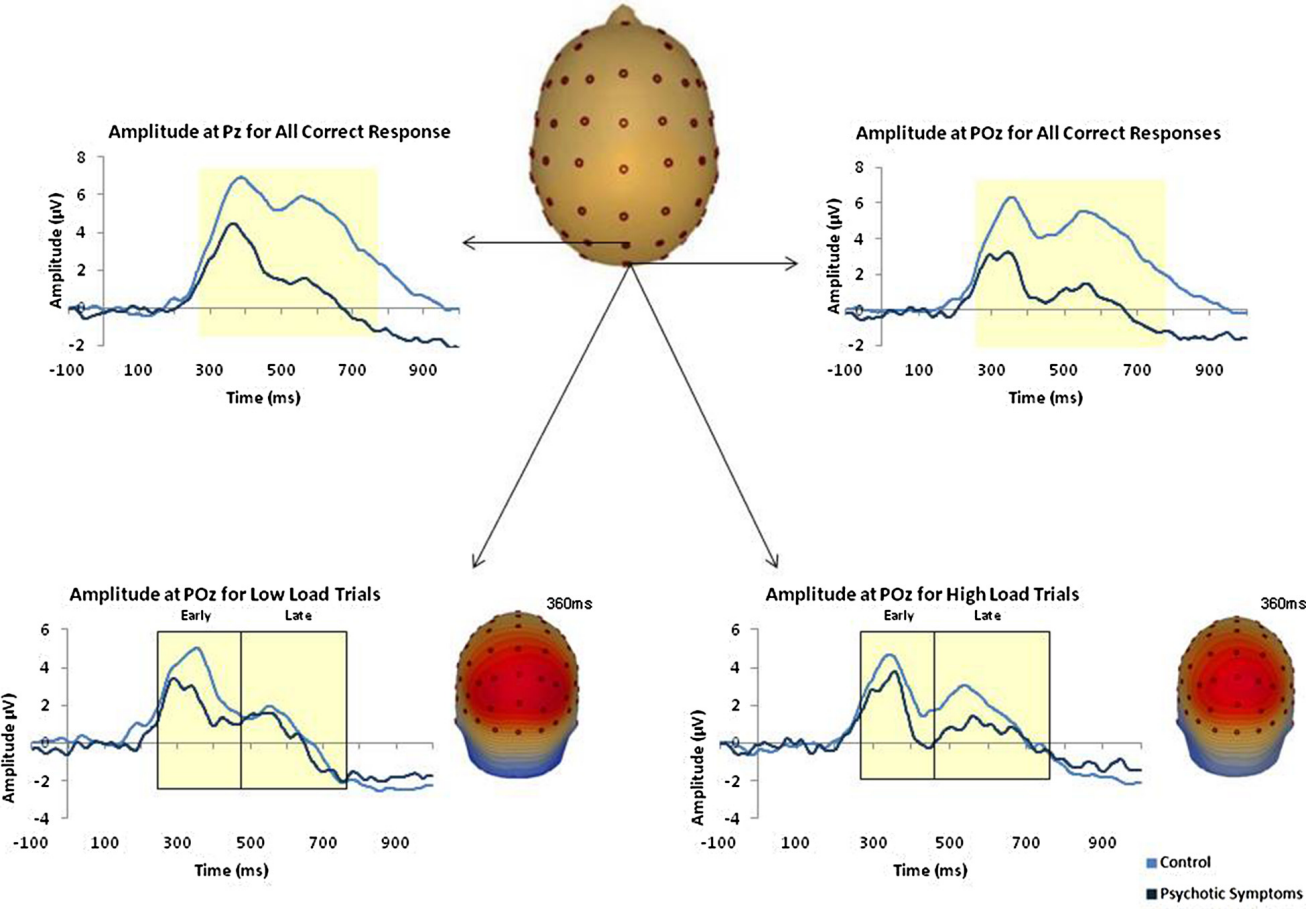
Accuracy (mean and S.E.M.) and reaction times (mean in ms and S.E.M.) for the spatial working memory task.

### Electrophysiological data

A between-groups effect was observed for mean amplitude at posterior electrode site Pz [*F*(1, 38) = 4.44, *p* = 0.04] for the timeframe 250 ms to 750 ms post-stimulus, with mean amplitude reduced in the psychotic symptoms group [Low Load: *M* = 4.15, *SEM* = 0.46; High Load: *M* = 3.57, *SEM* = 0.6] relative to the control group [Low Load: *M* = 4.94, *SEM* =0.37; High Load: *M* = 4.96, *SEM* =0.44]. A between-groups effect was also observed for mean amplitude at posterior electrode site POz [*F*(1, 38) = 7.53, *p* = 0.01], with mean amplitude reduced in the psychotic symptoms group [Low Load: *M* = 2.86, *SEM* = 0.55; High Load: *M* = 2.57, *SEM* = 0.68] group relative to the control group [Low Load: *M* = 4.42, *SEM* = 0.44; High Load: *M* = 4.61, *SEM* = 0.53]. For the same timeframe, no main or interaction effects of load or location were observed at electrode sites Pz or POz (see Figure 3).

Independent-samples t-tests revealed that when all correct responses were examined together, regardless of probe type, ERP amplitude at electrode site POz was reduced for both low load [*t*(40) = 2.21, *p* = 0.03] and high load [*t*(38) = 2.37, *p* = 0.02] for the timeframe 250 to 750 ms post-stimulus for the psychotic symptoms group compared to the control group. Significantly reduced mean amplitude was observed in the psychotic symptoms group relative to the control group at Low Load for Positive Probes [*t*(40) = 2.25, *p* = 0.03] and at High Load for Negative Probes [*t*(38) = 2.67, *p* = 0.01] at electrode site POz.

Due to the presence of two subcomponents of the P300, mean amplitude was assessed at Pz and POz for the timeframes 250 ms to 430 ms and 430 ms to 750 ms, respectively. A between-groups difference was observed at both electrodes [Pz: *F*(1, 38) = 4.11, *p* = 0.05; POz: *F*(1, 38) = 7.76, *p* = 0.01]. A Load * Time interaction [*F*(1, 38) = 7.18, *p* = 0.01] and a Load * Time * Group interaction were observed at POz [*F*(1, 38) = 5.68, *p* = 0.02]. Amplitude at POz was significantly reduced in the psychotic symptoms group at both early [250 ms to 430 ms; *t*(38) = 2.52, *p* = 0.02] and late time windows [430 ms to 750 ms; *t*(38) = 2.53, *p* = 0.02]. Further independent-samples t-tests revealed that mean amplitude for the psychotic symptoms group was reduced at low load for early [*t*(40) = 2.57, *p* = 0.01] and at high load for late [*t*(38) = 2.5, *p* = 0.02] time windows at POz. Mean amplitude was also reduced in the psychotic symptoms group at Pz for high load at the late timeframe [*t*(38) = 2.00, *p* = 0.05].

No main effect of load was observed for the latency of early P300 component at Pz [*F*(1, 38) = 0.01, *p* = 0.93] or POz[*F*(1, 38) = 0.61, *p* =0.44]. In addition, no between-group differences in the latency of the P300 component were observed at Pz [*F*(1, 38) = 0.1, *p* = 0.76] and POz [*F*(1, 38) = 0.97, *p* = 0.33] and no interaction effects were observed between load and group at either electrode site. In addition, mean reaction time was found to correlate negatively with mean amplitude at Pz at high load for the overall group [*r* = −0.4, *p* = 0.01; control: *r* = −0.326, *p* = 0.12; psychotic symptoms group: *r* = −0.48, *p* = 0.06]. This relationship was not observed at electrode site POz [overall group: *r* = 0.03, *p* = 0.86; control: *r* = 0.21, *p* = 0.32; psychotic symptoms group: *r* = −0.11, *p* = 0.69].

No between-groups effect on mean amplitude was observed at electrode site Fz for the timeframe 330 ms to 850 ms post-stimulus [*F*(1, 38) = 1.29, *p* = 0.26]. No main effects of load [*F*(1, 38) = 0.44, *p* = 0.51] or location [*F*(1, 38) = 1.35, *p* = 0.25] were observed, and no interaction effect [*F*(1, 38) = 2.58, *p* = 0.12] was observed at electrode site Fz for this timeframe.

## Discussion

The current paper reports reduced parietal positivity related to spatial working memory in a group of adolescents who reported psychotic symptoms compared to a control group using a computerised task based on the Sternberg working memory paradigm [38]. A sustained posterior positivity was observed in all conditions of the task with reduced P300 amplitude at posterior electrode sites in the psychotic symptoms group relative to the control group.

Although the groups were matched for accuracy and reaction times, subtle differences in the EEG data were observed which indicates that the PLEs group may have recruited difference brain regions or engaged in different cognitive strategies during the task despite performing at the same level as the control group, behaviourally. Mean reaction times were found to correlate negatively with mean amplitude at Pz at high load for the overall group, indicating those participants who took longer to respond at high load also had reduced amplitude at this posterior electrode site for the more difficult levels of the task. Although this correlation did not remain significant when each group was examined separately, the result for the overall group was mainly affected by a trend within the psychotic symptoms group (*p* = 0.06). When the P300 component observed at Pz and POz was divided and the early (250 ms to 430 ms) and late (430 ms to 750 ms) aspects of the component were examined separately reduced amplitude was observed in the psychotic symptoms group relative to the controls for both timeframes. Reduced amplitude was observed for low load at the early time window at POz and high load at the late time window at POz and also at Pz.

As anticipated, for the overall group, statistically significant decreases in accuracy scores and increases in mean reaction time were observed from low load to high load, i.e. as difficulty increased from Block 1 to Block 4 of the task. Accuracy and reaction time scores did not differ between the control and psychotic symptoms groups overall or at either low load or high load. Despite no statistically significant differences in the behavioural data, the pattern of responses indicates that at high load the psychotic symptoms group were slower to respond with greater deviation from the mean response time.

Reduced amplitude for the later part of the P300 component at high load indicates that, unlike the control group, the psychotic symptoms group did not show the same level of sustained parietal positivity to the probe at the more difficult levels of the task which other evidence supports [37]. The P300 ERP has been conceptualised as the physiological correlate of working memory updating of changes in the environment or as an index of allocation of attentional resources and memory search processes [35], [36]. Haenschel et al. [54] interpreted a decrease in the amplitude of the early P3b component during working memory retrieval in a group with early-onset schizophrenia to reflect a deficit in the evaluation of the probe stimulus against the stimulus representations held in memory. The reduced amplitude of early P300 observed in the present study may reflect disrupted neural processes underlying stimulus evaluation in the psychotic symptoms group which has not yet reached a level which would cause behavioural impairment. Reduced amplitude of the P300 component, particularly the late P300 at high load, could reflect an underlying reduced attention and memory retrieval capacity in the psychotic symptoms group. While impaired behavioural performance is commonly observed in patients with schizophrenia alongside abnormal neural responses to working memory tasks, we speculate that the disrupted neural responses observed in the PLEs group in the present study could reflect early changes in neuroanatomy which have not yet reached a level which would cause behavioural impairment on this task. Similarly, using a Go/No Go task with fMRI, Jacobson and colleagues [62] reported differences in patterns of brain activations in children reporting subclinical symptoms compared to a control group. Task related differences were noted in the absence of between-group differences in accuracy or reaction times in this study.

The current study adds to the previous literature by highlighting the P300 component and reductions in the amplitude of this component as a potential marker of psychotic symptoms in adolescence. Previous research by Murphy et al. [63] found reduced amplitude of the P300 component in a group of adolescents who reported psychotic symptoms compared to a control group on a receptive language task. Individuals with psychotic symptoms were characterised by reduced accuracy and smaller P300 amplitude on a computerised version of the British Picture Vocabulary Scale [64]. Similar to the present study, Murphy et al. [63] did not observe any between-group differences in the latency of the P300 component in their study. Previous research has suggested that increased latency of the P300 component in psychotic disorders is associated with increased age and illness duration [65].

A previous study by Jacobson et al. [62] found evidence of disrupted prefrontal-temporal connectivity in a group of adolescents reporting psychotic symptoms. Polich [40] hypothesised that the later P3b component may reflect attention and subsequent memory processes, and may originate from temporal-parietal activity associated with these processes. Evidence for parietal dysfunction at the earliest stages of psychosis has also been proposed by Whalley et al. [66]. Despite performing at a similar level to the control group behaviourally, the reduced P300 component may reflect a disrupted temporal-parietal network in the psychotic symptoms group.

Limitations of the present study include the small sample size and the poor spatial resolution of ERPs. In addition, an extensive IQ test was not administered and as a result the groups cannot be compared on this variable. This study used electrophysiological measures to investigate the neurophysiological underpinnings of spatial working memory in an adolescent sample with self-reported psychotic symptoms. This study is to our knowledge the first study to test spatial working memory in this sample using ERP measures. The present study therefore adds to the current literature by revealing reduced P300 amplitude in the treatment-naive extended psychosis phenotype. Further research is needed in order to uncover the relationship between spatial working memory performance and risk for psychotic disorder. The results of this study suggest that the neural networks involved in spatial working memory may be disrupted early in adolescence prior to the onset of a psychotic disorder.

## Conclusions

The present study expands existing findings of reduced P300 amplitude in adolescent onset schizophrenia [54] to the broader extended psychosis phenotype revealing reduced amplitude of the P300 ERP component in a community sample of adolescents who report psychotic symptoms during retrieval on a spatial working memory task. This reduction in P300 amplitude may reflect disrupted neural processes underlying stimulus evaluation and template matching during retrieval in the psychotic symptoms group. Reduced amplitude of the P300 component, particularly the late P300 at high load, observed in the present study could also reflect an underlying reduced attention and memory retrieval capacity in individuals with psychotic symptoms. These results identify neural correlates of neurocognitive dysfunction associated with population level psychotic symptoms and provide insights into ERP abnormalities associated with the extended psychosis phenotype. Follow-up studies may elucidate whether reduced P300 amplitude confers greater risk for developing a psychotic disorder in adulthood.

## Abbreviations

μV: Microvolts; ARMS: At-risk mental state; C-GAS: Children’s Global Assessment Scale; dlPFC: Dorsolateral prefrontal cortex; EEG: Electroencephalography; ERP: Event-related potential; FEP: First-episode psychosis; K-SADS-PL: Kiddie Schedule for Affective Disorders and Schizophrenia – Present and Lifetime version; PC: Parietal cortex; PFC: Prefrontal cortex; SEM: Standard error; UHR: Ultra high-risk; vlPFC: Ventrolateral prefrontal cortex; WRAT: Wide Range Achievement Test.

## Competing interests

The authors declare that they have no competing interests.

## Authors’ contributions

CR, MC, RR, MCC and MB designed the study. CR, JM, MB, MC and IK recruited the participant sample. CR set up and recorded the EEG, analysed the data and wrote the first and final drafts of the manuscript. JM, MB and FK helped carry out EEG set up and recording. IK conducted clinical interviews with the participants and their parents. RR aided the writing process and the data analysis. MCC and MC also helped to write and structure the manuscript. All authors read and approved the final manuscript.

## Pre-publication history

The pre-publication history for this paper can be accessed here:

http://www.biomedcentral.com/1471-244X/13/125/prepub
